# New High-Affinity Monoclonal Antibodies against Shiga Toxin 1 Facilitate the Detection of Hybrid Stx1/Stx2 *In Vivo*

**DOI:** 10.1371/journal.pone.0099854

**Published:** 2014-06-10

**Authors:** Craig Skinner, Stephanie Patfield, Larry H. Stanker, Pina Fratamico, Xiaohua He

**Affiliations:** 1 Western Regional Research Center, U.S. Department of Agriculture, Agricultural Research Service, Albany, California, United States of America; 2 Eastern Regional Research Center, U.S. Department of Agriculture, Agricultural Research Service, Wyndmoor, Pennslvania, United States of America; Wadsworth Center, New York State Dept. Health, United States of America

## Abstract

**Background:**

Shiga toxin-producing *E. coli* (STEC) are a group of common and potentially deadly intestinal pathogens expressing Shiga toxin (Stx) as a primary virulence factor. Of the two types of Stx, Stx2 is responsible for more severe symptoms during infection, while Stx1 is almost identical to the Shiga toxin from *Shigella dysenteriae*, a ubiquitous pathogen in developing countries. Although antibodies against Stx1 have been reported, few have reached the affinity needed for assembling highly sensitive immunoassays. Sensitive and affordable immunoassays for Stx1 and Stx2 could help improve detection of STEC in livestock, food, the environment, and in clinical samples resulting in improved food safety and human health.

**Method and Findings:**

Three new monoclonal antibodies (mAbs) against the B subunit of Stx1 were generated using recombinant toxoid Stx1E167Q and hybridoma technology. These new mAbs recognize all subtypes of Stx1, but do not cross-react with any subtype of Stx2. In addition, they exhibited the ability to neutralize Stx1 toxicity in Vero cell assays. An optimized sandwich ELISA using of a pair of these mAbs had a limit of detection of 8.7 pg/mL, which is superior to any existing assay of this kind. Using one of these Stx1 mAbs in concert with Stx2 mAbs, the presence of hybrid Stx1/Stx2 toxin in the culture media of STEC strains that express both Stx1 and Stx2 was demonstrated.

**Conclusions:**

These new mAbs provide a mix of availability, utility, versatility, and most importantly, increased sensitivity for detection of Stx1. There are numerous potential applications for these mAbs, including low-cost detection assays and therapeutic use. Analysis of hybrid Stx1/2 could provide new insights on the structure, activity, and cellular targets of Shiga toxins.

## Introduction

Shiga toxin-producing *Escherichia coli* (STEC) infections are a major health concern, and STEC is one of the most prevalent bacterial foodborne pathogens in developed countries, infecting more than 100,000 people each year in the United States alone [1]. Infections by members of the *Shigella* genus, especially *Shigella dysenteriae*, are very common in the developing world, and are estimated at approximately 90 million cases per year worldwide [2]. The clinical manifestations of STEC and *Shigella* infections range from diarrhea to hemmorhagic colitis and potentially deadly hemolytic uremic syndrome (HUS) [3]. These organisms share an important virulence factor: Shiga toxin (Stx in STEC; STx in *Shigella*), which is responsible for many of the worst symptoms of enterohemorrhagic *E. coli* (EHEC) and *Shigella* infections. All Stx sequences found in *E. coli* are thought to originate from horizontal gene transfer from the closely-related *Shigella* genus [4]. This gene transfer is likely to have been facilitated by lambdoid phages [5]. While the Stx-carrying phage is no longer capable of propagation in *Shigella* [6], many Stx-carrying phages in STEC are capable of lysis or lysogeny [4] which may ultimately disseminate Stx to even more species within the *Enterobactericiae* classification.

Although all Shiga toxins bind similar cellular receptors, the membrane glycolipids globotrioasylceramide (Gb3) and/or globotetraosylceramide (Gb4) [7], [8], and possess similar enzymatic activity (rRNA *N*-glycosidase) [9], there is considerable diversity within this class of toxins. Two main types of Stx are found in *E. coli*: Stx1, which is 98% identical to STx from the *Shigella* genus, and Stx2, which shares approximately 55% sequence identity with Stx1 and STx. There are numerous subtypes within the Stx1 and Stx2 types: three are recognized for Stx1 (Stx1a, Stx1c, and Stx1d), while seven are recognized for Stx2 (Stx2a through Stx2g) [10]. Stx1a and Stx2a are the prototypes of the Stx1 and Stx2 types, and are considered “wild type” Stx1 and Stx2. Stx subtypes vary in their toxicity as much as they do in their amino acid sequence. Although Stx1a may be slightly more toxic than Stx2a to Vero (African green monkey kidney) cells [11], Stx2a is much more toxic than Stx1a (more than 100-fold) to mice [12] and primates [13]. Among the Stx2 subtypes, Stx2a, Stx2c, and Stx2d are most commonly associated with severe human disease and HUS, while Stx2e-expressing strains of STEC can cause edema disease in piglets [14]. Stx1 is less frequently associated with HUS, and little is known about the toxicity of Stx1c or Stx1d [15], [16]. Stx1 and Stx2 can be found together in the same STEC strain as well, although it is unclear whether Stx1/Stx2 double expressing strains of STEC are as toxic as those expressing Stx2 alone [17]. Hybrid Stx1/Stx2 molecules have been generated using over-expression constructs [18], so it is possible that strains that express both Stx1 and Stx2 produce hybrid toxins as well, which might also play a role in toxicity.

Treating STEC infections is a very convoluted endeavor. Stxs (both Stx1 and Stx2, but not STx from *Shigella*) are encoded by phages and their expression is driven by a late-phase phage promoter, so whenever the bacterial host recognizes cellular stress, phage lysis genes and Stx genes are expressed. Unfortunately, antibiotics often cause cellular stress, and thus treating STEC with antibiotics, especially genotoxic antibiotics that induce the SOS response, can result in higher levels of Stx [19], and worsen symptoms of STEC infection [20]. The fluoroquinolones, especially ciprofloxacin, are the most notorious Stx-inducing antibiotics [21]. One of the more promising avenues for treatment of STEC, and possibly HUS as well, is by neutralizing anti-Stx antibody therapy [22]–[24]. Administration of the proper neutralizing antibodies (both anti-Stx1 and anti-Stx2, since they generally do not cross-protect) [25] could reduce the duration and severity of STEC infections.

Detection methods for Stx1 generally fall into two categories. PCR-based methods are extremely sensitive, but only detect the *stx1* gene encoding Stx1 or one of the Stx1 subtypes. Antibody-based methods are designed to detect the Stx1 molecule. Some antibody-based Stx1 detection kits can detect all three subtypes of Stx1 [26], [27]; however, they also cross react with some subtypes of Stx2. In addition, these antibodies generally are not available outside of their detection kits. There are several Stx1 antibodies commercially available separately from detection kits, but these antibodies are expensive, and assays using these antibodies are not overly sensitive. Here, we report the development of three high-affinity mouse mAbs against Stx1. Immunoassays using these new mAbs can detect low amounts of Stx1 (8.7 pg/mL). Additionally, we demonstrate that these antibodies are capable of protecting Vero cells from Stx1 toxicity, and, together with Stx2 antibodies, these mAbs were able to identify Stx1/Stx2 hybrids in vivo. The availability of these new mAbs will greatly improve cost-effective investigation on the prevalence of Stx1-producing STEC in food, the environment, and in clinical samples, and offer a potential treatment of HUS.

## Materials and Methods

### Ethics statement

All procedures with animals were carried out according to institutional guidelines for husbandry approved by the Institutional Animal Care and Use Committee of the U.S. Department of Agriculture, Western Regional Research Center (USDA IACUC). This specific procedure and protocol was reviewed and approved by the USDA IACUC (Protocol# 09-J-10). Mice were euthanized using rapid cervical dislocation to minimize suffering.

### *E. coli* strains and growth conditions

Strains expressing Stx2a (RM10638), Stx2b (RM7005), Stx2c (RM10058), Stx2d (RM8013), Stx2e (RM7958), Stx2f (RM7007), and Stx2g (RM10468) were grown as described [28]. Stx1-expressing strains, including Stx1a (RM13506, RM11768), Stx1c/Stx2b (AA1, FF6), and Sx1d (RM13149, II9) also were grown as previously described. Briefly, autoclaved LB medium was inoculated with a bacterial strain, which was grown overnight at 37°C at 150 rpm, and then inoculated at a 50-fold dilution into fresh LB medium supplemented with 50 ng/mL mitomycin C, and this culture was grown overnight in a 37°C shaking incubator. The cultures were centrifuged, and the supernatant was filter-sterilized (0.2 µm). All strains used in this study are listed in Table 1.

**Table 1:** Strains used in this study.

Strain	Other names	Serotype	Biomolecule expressed	Origin	Reference
RM10638		O157:H7	Stx2a	Cow (2009)	[33]
RM7005	EH250	O188:H12	Stx2b	Clinical	[33]
RM10058		O157:H7	Stx2c	Bird (2009)	[33]
RM8013		ND^a^	Stx2d	Cow (2008)	[33]
RM7988		ND^a^	Stx2e	Water (2008)	[28]
RM7007	T4/97	O128:H2	Stx2f	Feral pigeon	[33]
RM10468		ND^a^	Stx2g	Cow (2009)	[33]
25922 (ATCC)	Seattle, 1946	O6	No toxin	Clinical	
RM13506		O45	Stx1a	Human	[34]
RM11768		ND^a^	Stx1a		This study
RM13149		ND^a^	Stx1d		This study
II9		ND^a^	Stx1d		This study
AA1		ND^a^	Stx1c/Stx2b		This study
FF6		ND^a^	Stx1c/Stx2b		This study
RM1913		O157:H7	Stx2a	Human	[34]
RM2367		O157:H7	Stx1a/Stx2a	Human	[34]
RM6649		O157:H7	Stx1a/Stx2a	Human	[34]
RM7543		O157:H7	Stx1a/Stx2a	Human	[34]
RM9872		O145	Stx1a/Stx2a^b^	Cow feces	[34]
RM12788		O111	Stx1a/Stx2a	Human	[34]

a Not determined.

b RM9872 is PCR and ELISA positive for both Stx1a and Stx2a.

### Cloning, expression, and purification of Stx1 (E167Q) toxoid

To generated a non-toxic Stx1 toxoid, the E167Q point mutation was introduced into the *stx1* gene by mutagenic PCR [29]. The *stx1* (E167Q) toxoid gene was incorporated into the pQE-T7-2 vector by methods previously described [29], using the following primers (5′-GGAATTCCATATGAAAATAATTATTTTTAGAGTG-3′, 5′-CGTAAAGCTTGAGCTGTCAC-3′, 5′-GTGACAGCTCAAGCTTTACG-3′, 5′- CCGCTCGAGTCTTACTAACGAAAAATAACTTCGCTGAA-3′), and then transformed into BL21 (DE3) pLysS competent cells. Cells were grown in 250 mL of LB medium for 12 hours at 30°C and 150 rpm, and then induced with 1 mM IPTG (isopropyl β-D-1-thiogalactopyranoside, Sigma-Aldrich) overnight at 20°C, 150 rpm. The bacteria were centrifuged (5,000×g for 15 minutes in a Centrifuge 5430 R [Eppendorf, Hamburg, Germany] using the F-35-6-30 rotor) and the pellet was lysed by sonication (40% amplitude, 6×10-second pulses on a Sonic Dismembrator Model 500 [Fisher Scientific]). The debris was then removed by centrifugation (12,000×g, 10 minutes) and discarded. MnCl_2_ was added to the supernatant at a final concentration of 50 mM, and the mixture was incubated at RT for 10 minutes with stirring. The MnCl_2_ precipitate was removed by centrifugation (5,000×g, 30 minutes) and discarded. NH_4_SO_4_ was added to the supernatant to 70% saturation and the mixture was incubated on ice for 15 minutes while being stirred. The NH_4_SO_4_ precipitate was pelleted (5,000×g, 30 minutes), resuspended in PBS, and desalted using a Zeba spin column (10 kD pore size, Fisher). Stx1 (E167Q) was then purified using an anti-Stx1 B column (the 3C10 antibody [Toxin Technologies]) was conjugated to an amino-link column [Thermo Scientific]), dialyzed in phosphate buffered saline (PBS: 10 mM phosphate, 200 mM NaCl, pH 7.4), and filter sterilized (0.2 µm).

### Preparation of Stxs

Stx2 was purified from the Stx2a-expressing RM10638 strain as previously described [8]. Partially purified (approximately 14% pure: 0.5 mg/mL Stx1, 3.5 mg/mL total protein) Stx1 was purchased from Toxin Technologies (Sarasota, FL).

### Cell culture, immunization, and splenocyte extraction

SP2/0 myeloma cells and splenocytes were grown as previously described [28]. Mouse immunizations were conducted using Stx1 (E167Q) attenuated toxoid, suspended in the Sigma adjuvant system (Sigma-Aldrich). Each mouse received 5 µg of Stx1 (E167Q)/adjuvant mix by intraperitoneal injection at two-week intervals for a total of three injections. Two weeks after the third injection, mice were boosted with 1 µg/mouse Stx1 (E167Q) in sterile PBS. Three days later, mice were sacrificed by rapid cervical dislocation, spleens were excised, and splenocytes were harvested as previously described [28].

### Hybridoma development, cloning, and screening

Monoclonal antibodies (mAbs) were produced as described [28]. Cell fusions were achieved using SP2/0 myeloma cells, splenocytes extracted from the inoculated mouse spleen, and polyethylene glycol. The hybridomas were then subjected to three rounds of cloning by limited dilution, regrowth, and screening to isolate individual high-affinity antibody-producing hybridoma lines.

### Monoclonal antibody preparation

After clonal hybridoma lines were isolated, cells were grown in complete hybridoma medium (Iscove's modified Dulbecco's Minimal medium [Sigma-Aldrich] containing NaHCO_3_ [36 mM] and 1x Glutamax [Invitrogen, Carlsbad, CA], supplemented with 10% heat-inactivated fetal calf serum [FCS] [Invitrogen]). The purification of monoclonal antibody was conducted as described [28]. Briefly, 400 mL of antibody-containing media (hybridoma cells grown for 2–3 days) was passed through a Protein G column (GE Healthcare). Antibody was eluted with 0.1 M glycine (pH 2.7), resulting in 5–8 mg of purified Stx1 antibody. Protein concentration was determined using the BCA Protein Assay Kit (Thermo Scientific, Rockford, IL). Biotinylation of antibodies was performed using the Lightning-Link Biotin Conjugation Kit (Innova Biosciences, Cambridge, UK). Antibody isotyping was conducted by ELISA using Stx1 (E167Q) toxoid and horseradish peroxidase (HRP) -conjugated isotype-specific antibodies (Southern Biotech, Birmingham, AL).

### Commercial antibodies

Sifin 1 (VT 109/4-E9b, α-Stx1 B subunit), Sifin 2A (VT 135/6-B9, α-Stx2 A subunit), and Sifin 2B (VT 136/8-H4, α-Stx2 B subunit) were purchased from Sifin (Berlin, Germany). 13C4 (α-Stx1 B subunit), 3C10 (α-Stx1 A and B subunit, α-Stx2 A subunit), and PC1-HRP (“STXPC-1”, combination of 9C9, 3C10, 10D11, and BB12, HRP-conjugated) were purchased from Toxin Technologies.

### Enzyme-linked immunosorbent assays (ELISAs)

Hybridoma screening ELISAs were conducted as previously described [28]. Briefly, 50 ng/mL Stx1 (E167Q) toxoid, dissolved in PBS, was incubated overnight at 4°C in the wells of black Nunc Maxisorp ELISA plates. The plates were washed twice with PBS/0.05% Tween 20 (PBST), then blocking solution (5% nonfat dry milk/PBST) was added (200 µL/well), and the plates were incubated for 1 hour at RT. The plate was washed twice with PBST, then a combination of 50 µL/well blocking solution and 50 µL/well hybridoma medium was added, mixed, and incubated for 1 hour at RT. Following this, the plates were washed six times with PBST, and 100 µL/well of a 1/5,000 dilution of HRP-conjugated goat anti-mouse IgG antibody (GAM-HRP [Promega]) in blocking solution was dispensed into the plates, and incubated for 1 hour at RT. Following another six washes with PBST, 100 µL/well Pico chemiluminescent substate (Thermo Scientific) was added, and 5 minutes later, luminescence was measured using a Victor 3 plate reader (Perkin Elmer). All washes were conducted using a BioTek ELx405 plate washer. All ELISAs were conducted thrice, with the exception of the hybridoma screening ELISAs (once), and a representative ELISA is shown.

For sandwich ELISAs, coating antibody was diluted in PBS to 1 µg/mL, and then 100 µL/well was allowed to bind to black Maxisorb plates overnight at 4°C. This was followed by washing twice with PBST, and then 200 µL/well blocking solution (3% BSA in PBST) was added and incubated for 1 hour at RT. The indicated toxin (Stx1 or Stx2) or a 20-fold dilution of cell-free medium, diluted in PBS, was then added at 100 µL/well and incubated at RT for 1 hour. The plates were then washed six times with PBST, then 100 µL/well of the indicated biotinylated secondary antibody (0.25 µg/mL, diluted in BSA/PBST) was added and incubated for 1 hour at RT. The plates were washed a further six times and 100 µL/well of 0.2 µg/mL streptavidin-HRP (SA-HRP) (diluted in BSA/PBST) was added, and the plates were incubated for 1 hour at RT. Following this, the plates were washed a final six times and developed using 100 µL/well of Pico chemiluminescent substrate (Thermo Scientific). Luminescence was measured using a Victor 3 plate reader. All sandwich ELISAs were conducted thrice for confirmation. Limit of detection (LOD) was calculated by extrapolating ng/mL of Stx1 (E167Q) from the background luminescence plus 3 standard deviations of the background.

### Western blots

Western blots were conducted as described [8]. Pure protein and cell-free medium samples were incubated at 72°C for 10 minutes in 1x NuPage SDS loading buffer, then run on a 4%–12% NuPAGE Novex Bis-Tris mini gel (Invitrogen). The proteins were then transferred to a PVDF membrane (pore size, 0.45 µm; Amersham Hybond-P), blocked with 2% ECL Prime blocking agent (GE Healthcare) in PBST for 1 hour at RT, and washed three times with PBST (3 minutes each). Monoclonal antibodies were diluted to 1 µg/mL in blocking solution and incubated with the blots for 1 hour at RT, then the blots were washed thrice again in PBST. GAM-HRP antibody (Promega) at a 1/10,000 dilution was incubated on the blot for 1 hour at RT. The blots were washed four more times with PBST (5 minutes each), and developed using Lumigen TMA-6 (Lumigen) substrate. The blots were visualized with a 5 minute exposure using a FluorChem HD2 (Alpha Innotech). All westerns were conducted three times.

### Anibody affinity measurements and isotyping

Antibody affinity to Stx1 (E167Q) was measured using an Octet QK system (Forte-bio, Menlo Park, CA) as previously described [28]. Briefly, biotinylated mAbs were bound to streptavidin biosensors at 10 µg/mL, diluted in PBS. Stx1 (E167Q) was then incubated with the sensors at four different concentrations (142, 71, 36, and 18 nM) and then allowed to dissociate in PBS. Dissociation constants (K_D_) were calculated using the Octet QK software (Data Acquisition 7.0). Antibody isotyping was conducted by ELISA using an isotype-specific antibody panel (Southern Biotech, Birmingham, AL). Stx1 (E167Q) at 500 ng/mL and 100 µL/well was coated onto a clear Nunc Maxisorp ELISA plate overnight at 4°C. The plate was washed twice with PBST, then blocked with 5% nonfat dry milk/PBST for one hour at RT (200 µL/well). The plate was washed twice more, and 100 ng/mL mAb was added (100 µL/well) for one hour at RT. The plate was then washed six times, then HRP-conjugated isotype-specific antibody was added at a 1/1000 dilution (100 µL/mL) and the plate was incubated for one hour at RT. The plate was washed a final six times, TMB substrate was added at 100 µL/well, and after 3 minutes 100 µL/well 0.3 N HCl was added. Absorbance was then read at 450 nm on the Victor 3 plate reader.

### Vero cell cytotoxicity assays

Vero cells were prepared and grown as described [28]. Briefly, the medium used for Vero cell propagation and growth was Dulbecco's Modified Eagle Medium (DMEM, Invitrogen) with 10% fetal bovine serum (FBS) (Invitrogen), within a humidified cell culture incubator (37°C, 5% CO_2_). Before conducting the cytotoxicity assays, the cells were trypsinized, diluted to 10^5^ cells/mL, then dispensed into 96-well cell-culture-treated plates, and these plates were incubated for 24 hours. Sterile purified Stx or filtered-sterilized mitomycin-induced STEC culture medium was diluted in fresh Vero media (1 ng/mL final concentration for purified Stx; 1 µL media/well for cell-free medium). mAbs were pre-incubated at the stated concentrations with toxin for 1 hour at RT before adding the mixture to the Vero cells. The medium in the Vero cell assay plate was then removed and replaced with the Stx- or STEC medium-containing mixture (100 µL/well), with or without antibodies. Twenty-four hours after treatment, the Vero cells were lysed using 100 µl/well of CellTitre-Glo reagent (Promega) diluted 1/5 in PBS, with 3 minutes of shaking. Luminescence was measured using a Victor II plate reader. All Vero cell toxicity assays were conducted three times with similar results.

## Results

### Isolation and characterization of anti-Stx1 monoclonal antibodies

**Figure 1:**
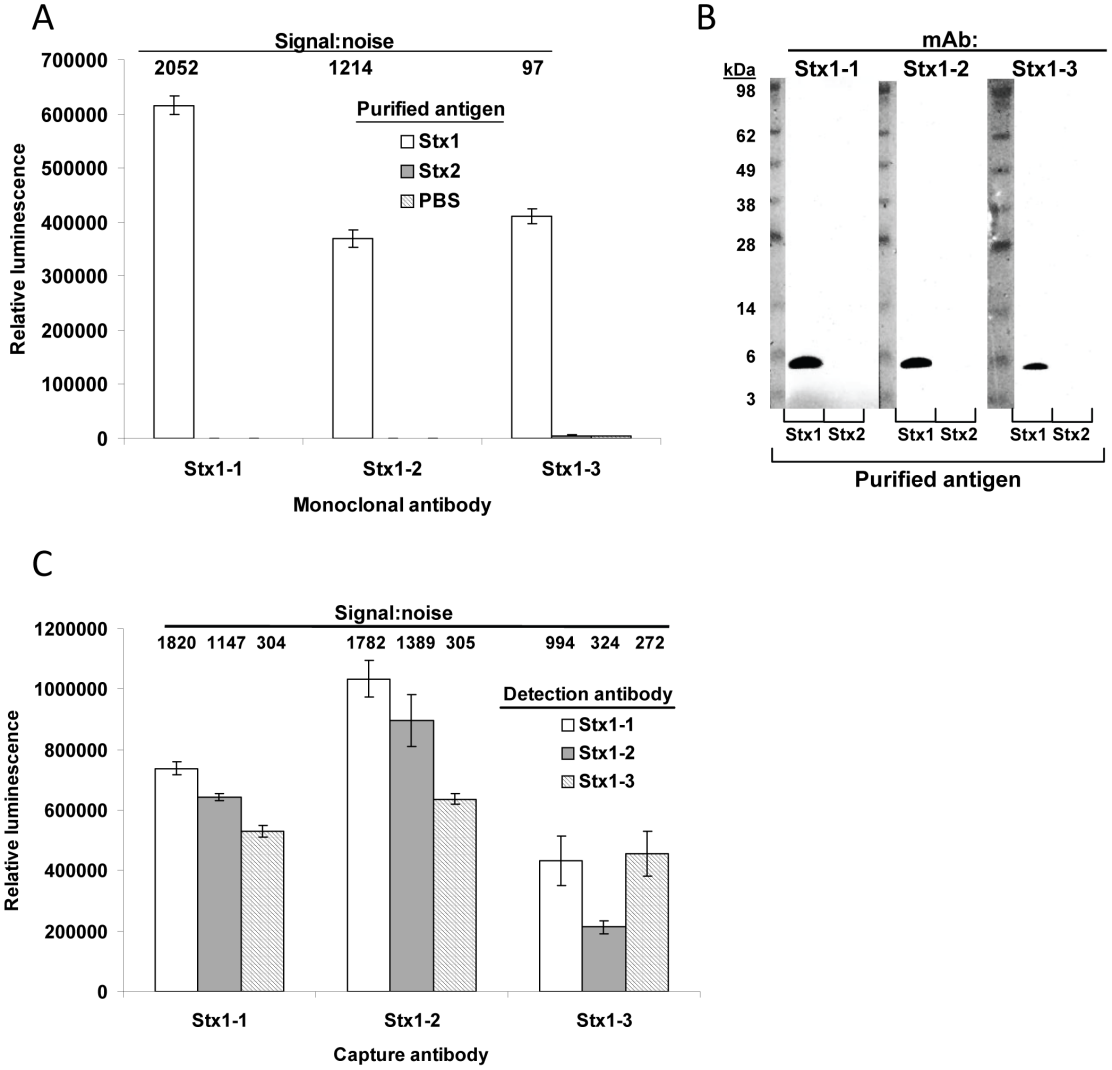
Antigen specificity of the Stx1 mAbs. A. Direct ELISAs using new Stx1 mAbs and partially purified Stx1 (from Toxin Technologies) and purified Stx2. Stx1 mAbs were used at 100 ng/mL and GAM-HRP at a 1/5000 dilution. B. Western blots using the Stx1 mAbs and purified or crude toxin preparations. Purified (Stx2) and partially purified (Stx1) toxin was added at 50 ng/lane. C. Sandwich ELISA antibody combination optimizations. Partially purified Stx1 (from Toxin Technologies) was used for these assays, at a concentration of 100 ng/mL.

A recombinant catalytically inactive mutant (E167Q) toxoid of Stx1 was expressed in *E. coli* (a listing of all bacterial strains used in this study is included in Table 1), purified by antibody affinity, and injected into mice. Using standard hybridoma techniques, splenocyte-myeloma fusions were prepared, and a total of 960 wells of hybridomas were screened for Stx1 recognition. After repeated cloning and recovery steps, three high-affinity antibody-producing hybridoma lines were isolated. All three of these mAbs (mAb Stx1-1, Stx1-2, and Stx1-3) have exclusive specificity to Stx1 and do not react with Stx2 in direct ELISA assays (Figure 1A). Even though the native form of the Stx2 B subunit is considered to be less immunogenic than the A subunit [29], all three Stx1 mAbs recognized the B subunit (Figure 1B). All three antibodies have kappa light chains and belong to different IgG subtypes (IgG2a for Stx1-1, IgG1 for Stx1-2, and IgG2b for Stx1-3), and all three have low K_D_ values (below 10^−9^ M) (Table 2). Although all combinations of antibody capture/detection pairs could detect Stx1, those using mAbs Stx1-1 and Stx1-2 (particularly Stx1-2 as capture antibody and Stx1-1 as detection antibody) had both the strongest signals and highest signal:noise ratios (Figure 1C).

**Table 2:** Properties of Stx1 monoclonal antibodies.

Antibody	Isotype	K_D_ (x 10^−9^ M)
Stx1-1	IgG2a, kappa	0.437
Stx1-2	IgG1, kappa	0.704
Stx1-3	IgG2b, kappa	0.0255

### Sensitivity and subtype-specificity of ELISAs utilizing the mAb combination of Stx1-2/Stx1-1

**Figure 2:**
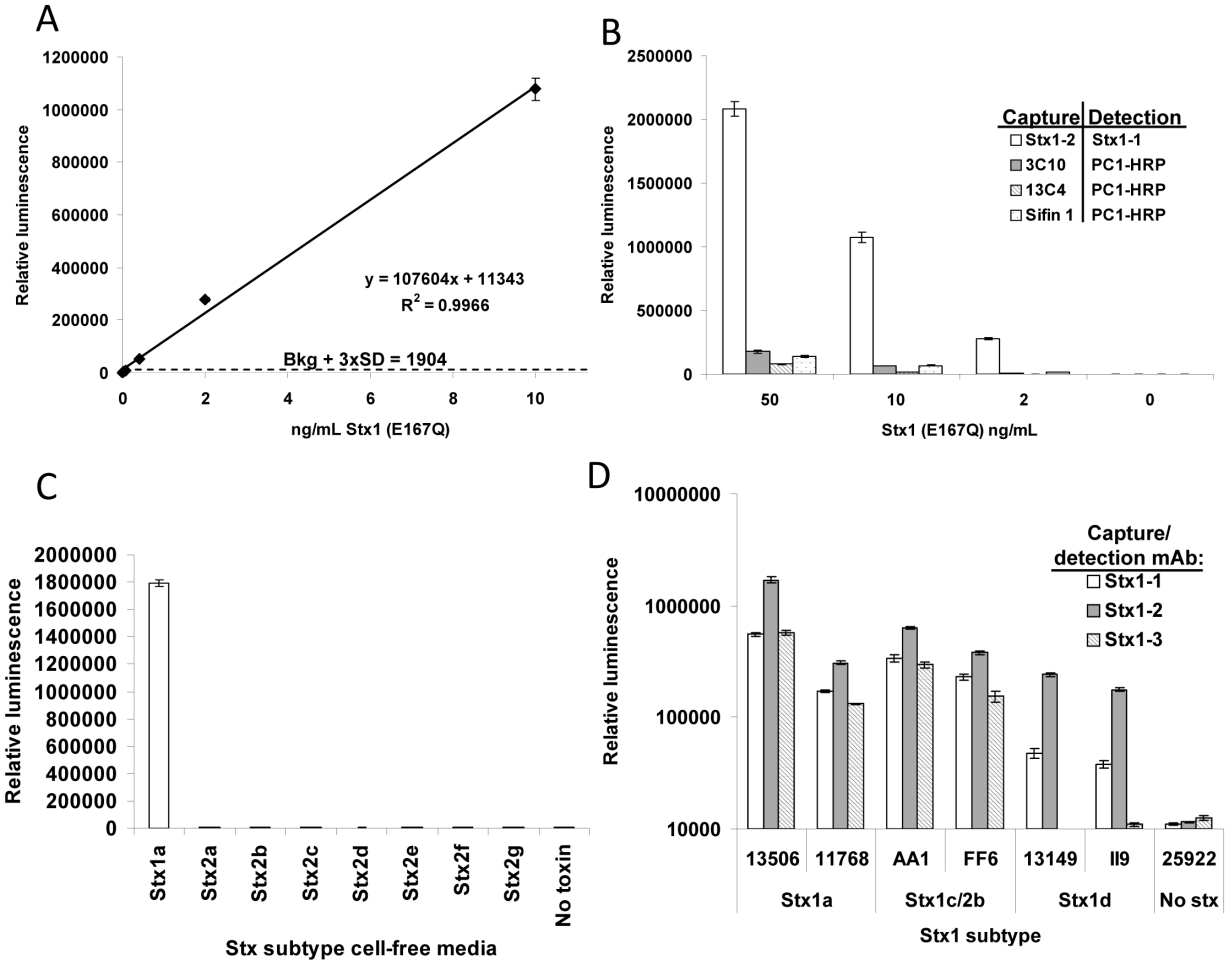
Stx1 ELISA sensitivity and subtype specificity. A. Standard curve for an anti-Stx1 sandwich ELISA using mAbs Stx1-2 and Stx1-1. Purified Stx1 (E167Q) toxoid is used as the antigen; mAb Stx1-2 as the capture antibody (at 1 µg/mL), and mAb Stx1-1-biotin as the detection antibody (at 0.25 µg/mL). The standard curve was linear from 10 to 0.01 ng/mL (R^2^ = 0.997). B. Comparison of the mAb Stx1-2/Stx1-1 ELISA with commercially-available antibodies. The anti-Stx1 antibodies 3C10 (against the A subunit), 13C4, and Sifin 1 (both against the B subunit) were used for capture while PC1-HRP was used for detection. C. Cross-reaction of the Stx1 ELISA (Stx1-2/Stx1-1) with Stx2 subtypes. Experiment was conducted using a 1/20 dilution of cell-free medium (induced with 50 ng/mL mitomycin C). D. Stx1 subtype specificity of the mAbs using a single monoclonal antibody sandwich ELISA. Relative luminescence is shown in the log scale on the Y-axis.

The limit of detection (LOD) for pure Stx1 in PBS by the sandwich ELISA using mAbs Stx1-2 as a capturing antibody and Stx1-1 as a detecting antibody was 8.7 pg/mL (Figure 2A), almost nine-fold more sensitive than that indicated on the package insert of the EHEC Premier kit (Meridian) 70 pg/mL. To evaluate our mAbs, we compared the performance of our antibodies to commercially available Stx1 antibodies by ELISA. For ELISAs using commercial antibodies, mAbs 3C10 (Toxin Technologies), 13C4 (ATCC Bioresource Center) and Sifin 1 (Sifin) were used as capture antibodies, respectively, and PC1-HRP (Toxin Technologies), which is a combination of Stx1 and Stx2 antibodies conjugated to HRP, was used as the detection antibody. Our most sensitive ELISA, using a combination of mAbs Stx1-2 (to capture) and Stx1-1 (to detect) was over 10-fold more sensitive than any of the three ELISAs using commercially available antibodies (Figure 2B), representing a considerable improvement.

Stx1 subtypes (Stx1a, 1c, and 1d) are very similar by amino acid sequence, while Stx2 subtypes (Stx2a through 2g) are more diverse, both in the A and B subunits. Although these three Stx1 mAbs do not recognize Stx2a, we tested for reactivity to the various Stx2 subtypes. We performed mAb Stx1-2/Stx1-1 sandwich ELISAs testing all seven Stx2 subtypes present in bacterial culture media. None of the Stx2 subtypes were recognized in these tests, reaffirming the assay's specificity to Stx1 (Figure 2C). The presence of Stx2 in these media was confirmed by Vero cell assays [28]. To determine whether the three mAbs can recognize all subtypes of Stx1, we used culture supernatants from STEC strains containing Stx1a (RM13506, RM11768), Stx1c/Stx2b (AA1, FF6), and Stx1d (RM13149, II9) as toxin sources and performed sandwich ELISA using a single mAb for both capture and detection, with the detection antibody being biotinylated. mAbs Stx1-2 and Stx1-1 could detect all three subtypes (Figure 2D), but mAb Stx1-3 could only effectively detect Stx1a and Stx1c. Among the three mAbs, mAb Stx1-2 was the most effective in binding all three subtypes of Stx1 based on the ELISA results (Figure 2D), but even using this antibody, the detection signal for Stx1d was 3 to 30-fold lower than that of Stx1a. This may suggest that mAb Stx1-2 does not recognize Stx1d as well as Stx1a, or it may simply indicate that the Stx1d-expressing strains release less toxin than the Stx1a strains.

### Neutralization of Stx1 cytotoxicity

**Figure 3:**
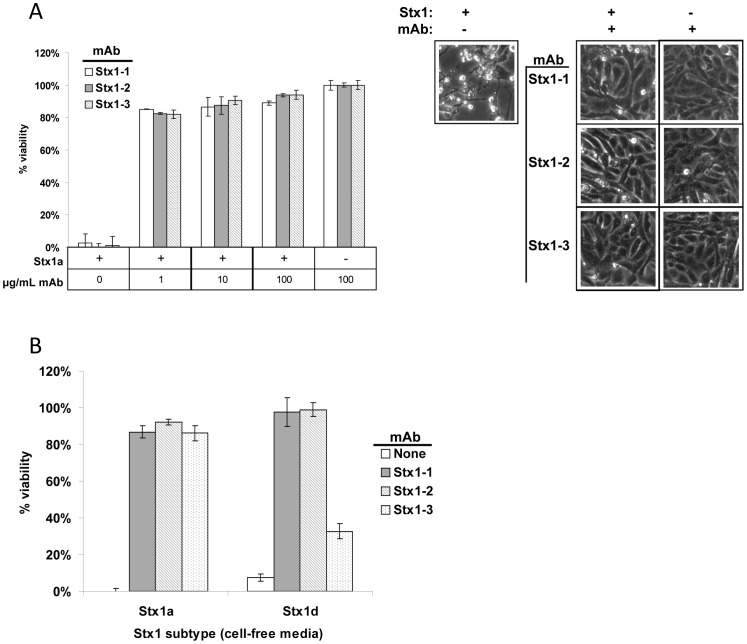
Neutralization of Stx1 toxicity by Stx1 mAbs. A. Administration of mAbs Stx1-1, Stx1-2, or Stx1-3 at 1, 10, or 100 µg/mL fully protects Vero cells from Stx1 toxicity (partially pure toxin at 1 ng/mL). Photographs are of Vero cells treated with 1 ng/mL Stx1 and 100 µg/mL mAb. B. mAbs Stx1-1 and Stx1-2 (at 100 µg/mL) protect Vero cells from Stx1a- and Stx1d-containing cell free media (13506 and 13149, respectively, 1 µL toxin-containing medium per well). mAb Stx1-3 (at 100 µg/mL) fully protects Vero cells from Stx1a but not Stx1d.

Our previous results showed that antibodies recognizing the Stx B subunit typically have strong toxin neutralizing activity *in vitro* [29]. In this study, all three mAbs were able to protect Vero cells from Stx1a toxicity (1 ng/mL toxin from Toxin Technologies) at an antibody concentration of 1, 10, or 100 µg/mL, although at 1 µg/mL, all three mAbs only neutralized around 80% of the toxin (Figure 3A). Since mAbs Stx1-1 and Stx1-2 also detected Stx1d in ELISA, we evaluated whether these mAbs can neutralize this subtype in cell-free medium, as well. Similar to ELISA results, mAb Stx1-3 was less effective at neutralizing Stx1d (Figure 3B). Stx1c was not included in the cell toxicity and neutralization assays since the strains we possess that express Stx1c also express Stx2b, and even if Stx1c is neutralized, Stx2b can kill the Vero cells.

### Detection of Stx1/Stx2 hybrid toxin *in vivo*

**Figure 4:**
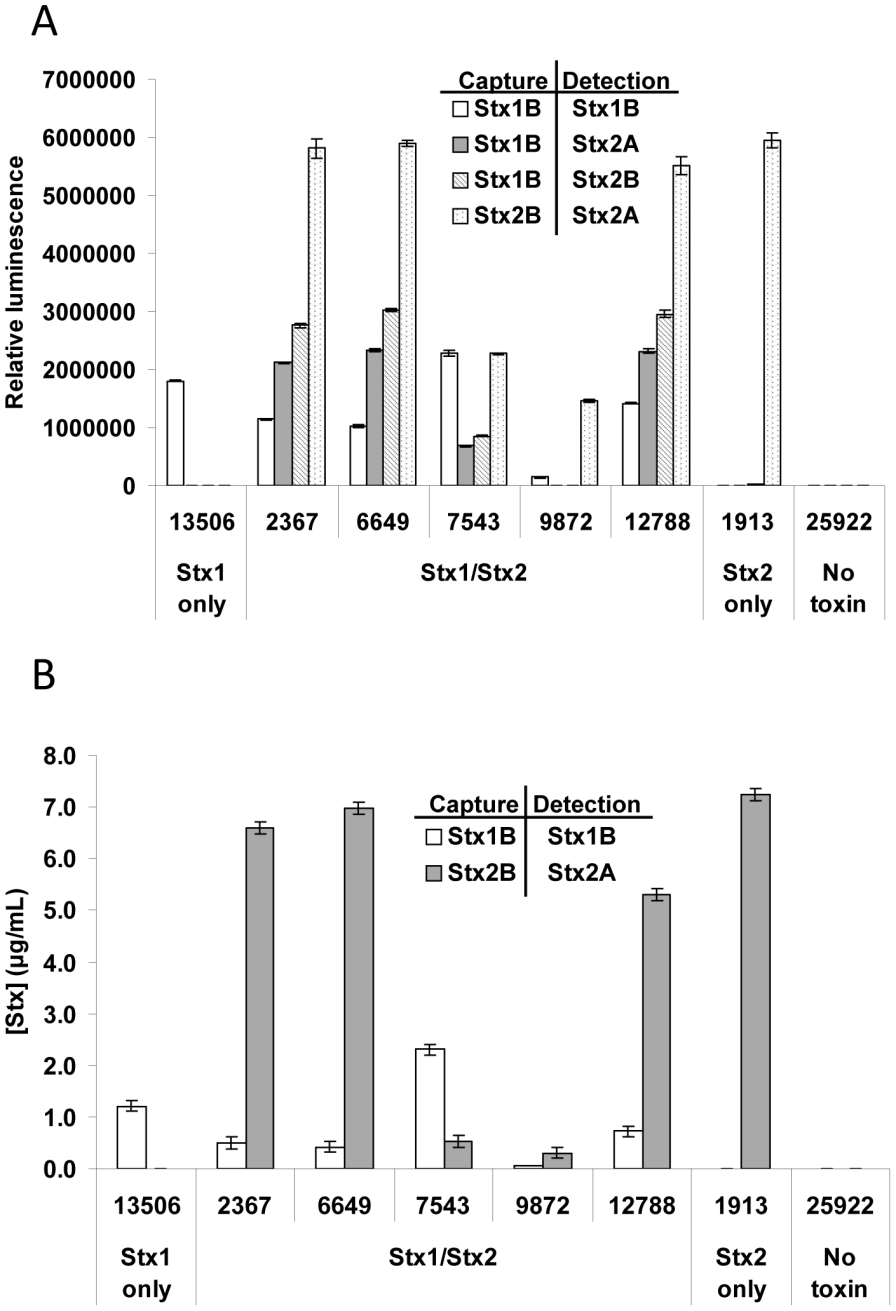
Detection of hybrid Stx1/Stx2. A. Using a Stx1 capture antibody and a Stx2 detection antibody, hybrid Stx1/2 molecules were detected by ELISA. Cell-free medium from mitomycin-induced STEC strains containing Stx1, Stx2, both Stx1 and Stx2, or neither was applied to the ELISA at 1/20 dilution in PBS (5 µL medium/well). B. Stx concentration was determined for the medium used in Figure 4A by ELISA, using a standard curve of Stx1 (E167Q) for Stx1 or purified Stx2a for Stx2.

It was reported that Stx1 and Stx2 subunits were capable of co-assembling into hybrid Stx molecules *in vivo* when over-expressed in *E. coli* [18], [30]. Using cultures of five isolates of STEC that express both Stx1 and Stx2, we sought to determine if hybrid Stx molecules exist naturally *in vivo* as well. We used mAb Stx1-2 as a capture antibody and Sifin 2A, which recognizes the A subunit of Stx2 and does not cross-react with Stx1 (Figure S1), as a detection antibody, assuming that this combination of antibodies would detect only hybrid toxin composed of one or more Stx1 B subunit and a Stx2 A subunit. Indeed, in the filter-sterilized culture media of four out of five of the Stx1/Stx2-expressing isolates tested, hybrid Stx was detected (Figure 4A, Stx1B capture/Stx2A detection). We also analyzed the abundance of hybrid B pentamers, using mAb Stx1-2 as a capture antibody and Sifin 2B (which recognizes the B subunit of Stx2 [9], but also weakly recognizes the A subunit of Stx2 [Figure S1]) as a detector. All strains which expressed both Stx1 and Stx2 also possessed considerable amounts of B subunit hybrids (Figure 4A, Stx1B capture/Stx2B detection), with the exception of Strain RM9872, according to this ELISA. Notably, no hybrids (Stx1B/Stx2A and Stx1B/Stx2B) were detected in control strains expressing Stx1 (RM13506) or Stx2 (RM1913) alone. We then used standard curves of Stx1a (purified E167Q) and Stx2a (purified toxin) to estimate the concentrations of these toxins in each media sample (Figure 4B). In general, the concentrations of Stx1 were much lower than that of Stx2 in bacterial culture media. However, we detected more Stx1 than Stx2 in the culture supernatant of RM7543. The amount of hybrid toxin appeared to be related to the relative expression of Stx1 and Stx2. In strains with high levels of both Stx1 and Stx2 (RM2367, RM6649, and RM12788), more hybrid toxin was observed. In strain RM9872, which expressed both Stx1 and Stx2 at very low levels, no hybrid toxin was evident. Due to the lack of a monoclonal antibody that recognizes only the Stx1 A subunit (3C10 may also recognize the B subunit of Stx1 and Stx2 [Toxin Technologies]), a hybrid Stx ELISA using mAbs against the Stx1 A subunit and the Stx2 B subunit was not attempted.

## Discussion

In this study, we report the development and characterization of three novel high-affinity monoclonal antibodies against Stx1. These three antibodies are independent and recognize only the B subunit of Stx1, and no apparent cross-reaction with any subtypes of Stx2 was observed. Two of the three mAbs (Stx1-1 and Stx1-2) recognized all three subtypes of Stx1 present in bacterial culture media; mAb Stx1-3 recognized only Stx1a and Stx1c, but not Stx1d, suggesting lower affinity of this mAb to Stx1d compared to the other two mAbs. The sandwich ELISA composed of mAb Stx1-2 (as capture antibody) and Stx1-1 (as detection antibody) was the most sensitive with a LOD of 8.7 pg/mL. mAbs Stx1-1 and Stx1-2 were able to fully neutralize the toxicity from both Stx1a and Stx1d. mAb Stx1-3 neutralized Stx1a as well, but only partially neutralized Stx1d, using the same antibody concentration (100 µg/mL). Using mAb Stx1-2 combined with an anti-Stx2 A subunit antibody, we then developed an ELISA capable of detecting Stx1/Stx2 hybrid toxins *in vivo*. Remarkably, we found hybrid toxins in four out of the five Stx1/Stx2 double-expressing strains tested. The one strain in which we did not detect hybrid toxins had relatively low levels of both Stx1 and Stx2.

The three subtypes of Stx1 are strongly conserved at the amino acid level [10]. There are only three positions where the mature B subunits of Stx1a, Stx1c, and Stx1d differ: (counting from the beginning of the mature protein) at amino acids 1, 25, and 55. Of these three sites, Stx1d differs from Stx1a and Stx1c only at amino acid 25 (G for Stx1a and 1c; A for Stx1d) and 55 (N for Stx1a and 1c; T for Stx1d). Of these two sites, amino acid 55 is the least conserved. Therefore, it is likely that the epitope for mAb Stx1-3, which recognizes Stx1a and Stx1c but not Stx1d, spans amino acid 55, and that this residue is important for epitope binding. STx from *Shigella spp.* and Stx1a are almost perfectly conserved at the amino acid level: they differ by only one amino acid in the A subunit. The B subunits of Stx1a and STx are identical, however, suggesting that the new mAbs will recognize STx as well, and with around an 8.7 pg/mL limit of detection. These antibodies, and the assays that incorporate them, should therefore be just as effective at detecting and neutralizing STx as Stx1a.

Since monoclonal antibodies recognize only one epitope, it is usually not possible to develop a sandwich ELISA with only one monoclonal antibody. However, our previous study indicates that two of our mAbs against Stx2, mAb Stx2-2 and Stx2-5 [29], recognize both the A and B subunits of Stx2, and an ELISA using the same mAb for capture and detection was functional (unpublished data). In this study, we noticed that all three ELISAs using a single mAb for both capture and detection were not only functional, but also highly sensitive. Moreover, these three antibodies were specific only to the B subunit of Stx1, and did not cross react with the A subunit (Figure 1B). These results suggest that a B subunit-specific mAb could serve as both capture and detector for an AB_5_ toxin in sandwich ELISAs. If one of the five B subunits is immobilized by the capture antibody, there are still 4 B-subunits available for the detection antibody. It is conceivable that a B subunit-targeting antibody is superior to an A-subunit antibody in terms of sensitivity as well since it has five times the epitopes to bind to. We can extend this observation not only to all AB_5_ toxins, but to potentially all stable homomultimeric proteins. Additionally, for a single mAb sandwich ELISA to work, the B subunit of the AB_5_ toxin must be assembled into a multimer: at least a dimer, but likely a pentamer. So a single mAb ELISA additionally provides information on a protein's quaternary structure, and this is another principle that may be applied to any stable homomultimeric protein. Of course, the success of a Stx1 B subunit single mAb sandwich ELISA does not confirm the presence of an A subunit, or a functional toxin.

Observation of Stx1/Stx2 hybrid toxins in cell-free media of bacterial strains from environmental STEC samples raises many questions as to the nature and functionality of these hybrid molecules. Previous studies have shown that the B subunit pentamer of a hybrid Stx molecule determines its cellular target and cytotoxicity [30]. However, those studies were conducted on cell culture lines (MRC-5 and Vero cells), where Stx1 may be more toxic than Stx2, unlike in mice, where Stx2 is vastly more lethal (>100-fold) than Stx1. If Stx1/Stx2 hybrid toxin is generated during STEC infections, what role does it play in toxicity? What would its toxicity, if it has any, be in mice or humans? And are Stx hybrids contributing to the virulence of double expresser strains or inhibiting it? Furthermore, expression of Stx1 often differs from that of Stx2. Stx1 tends to be more cell-associated, and is inducible by low levels of iron as well as by antibiotics that initiate the SOS response [31]. The concentration and composition of hybrid Stx molecules found in cell-free media may therefore be different from those found in cell lysate or cell-associated fractions. Additionally, under conditions of low iron but not genotoxic stress, Stx1 may figure more prominently in Stx hybrids than Stx2. Although hybrid Stx may not be more toxic than pure Stx1 or Stx2, it could have a wider range of cellular targets. As many as three or as few as one Gb3 molecules can bind each Stx B subunit [32]. Although the B subunits of Stx1 and Stx2 generally bind to Gb3, those of Stx2e and Stx2f have affinities for both Gb3 and Gb4 [7], [8]. Mixed pentamers containing a B subunit of Stx2e or Stx2f might be capable of binding both Gb3 and Gb4, and both of these receptors are found clustered in the lipid rafts of cells which express them. Regardless, the observation of Stx1/Stx2 hybrid toxins *in vivo* will help us better understand the mechanism of human infection by STEC.

With high sensitivity and exclusive specificity to Stx1 subtypes, these new mAbs can be easily incorporated into Stx1 detection methodologies. Additionally, they have high utility for research, as we have demonstrated by using them to detect hybrid Stx1/2 *in vivo*. We hope that these new Stx1 mAbs will help facilitate affordable Stx1 detection, and provide a convenient alternative to expensive detection kits for those who cannot afford them.

## Supporting Information

Figure S1 **Antibody specificities of Sifin 2A and Sifin 2B.** Sifin 2A recognizes only the A subunit of Stx2. Sifin 2B primarily recognizes the B subunit of Stx2, but also weakly detects the A subunit of Stx2. Mitomycin-induced (50 ng/mL) cell-free supernatants (13 µL/lane) were used in this Western blot: 13506 for Stx1, 1913 for Stx2, and 25922 as a negative control.
(TIF)
