# Supplementary figures and images for: New High-Affinity Monoclonal Antibodies against Shiga Toxin 1 Facilitate the Detection of Hybrid Stx1/Stx2 In Vivo
Source: PLoS One. 2014 Jun 10;9(6):e99854. doi: 10.1371/journal.pone.0099854 (PMC4051773; doi:10.1371/journal.pone.0099854)

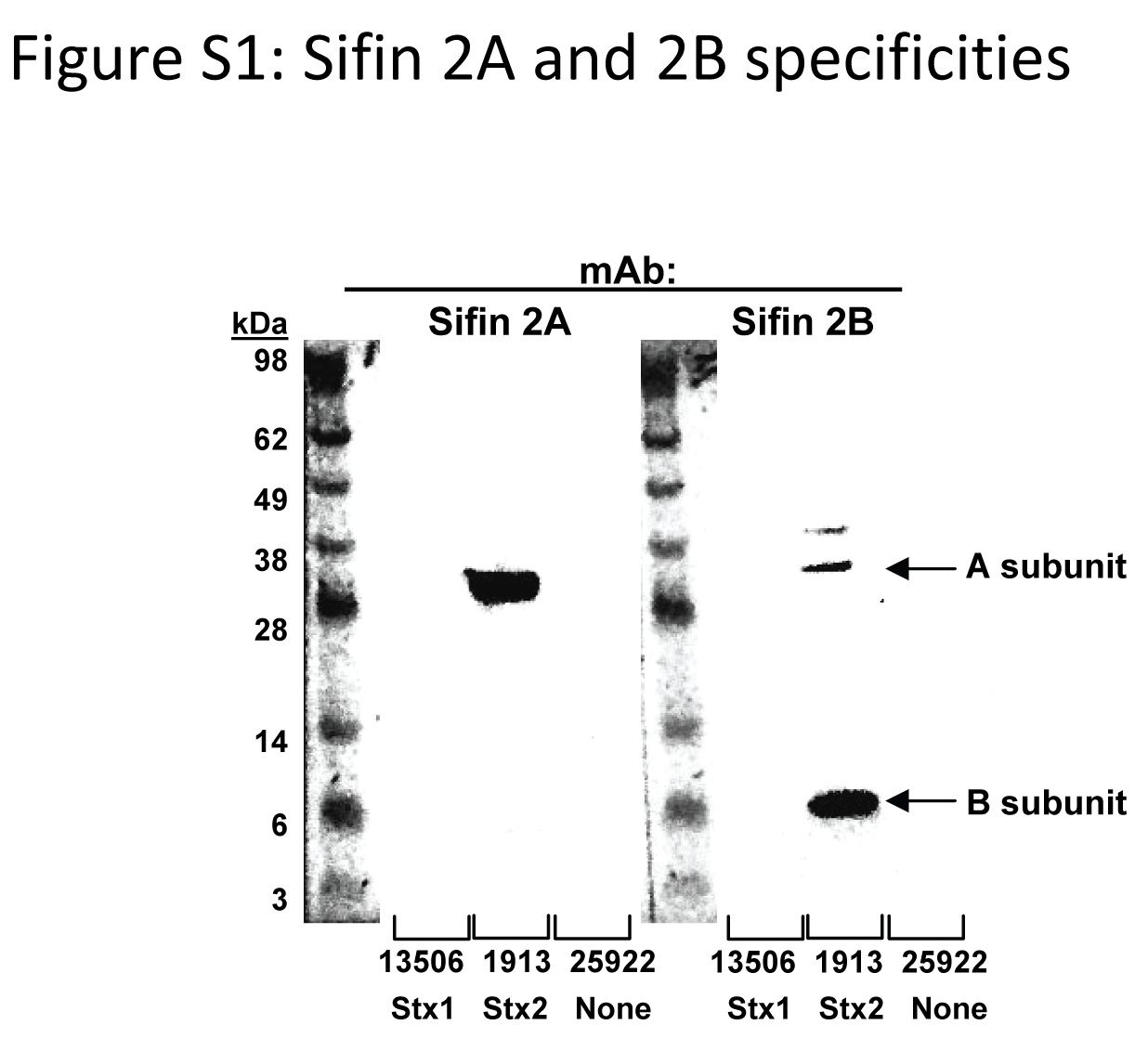

Supplement: Figure S1 — Antibody specificities of Sifin 2A and Sifin 2B. Sifin 2A recognizes only the A subunit of Stx2. Sifin 2B primarily recognizes the B subunit of Stx2, but also weakly detects the A subunit of Stx2. Mitomycin-induced (50 ng/mL) cell-free supernatants (13 µL/lane) were used in this Western blot: 13506 for Stx1, 1913 for Stx2, and 25922 as a negative control. (TIF) [file pone.0099854.s001.tif]
